# 825. Hyperbaric Oxygen Therapy in Burn Care: A Systematic Review of Current Evidence

**DOI:** 10.1093/jbcr/irag033.264

**Published:** 2026-04-06

**Authors:** Julissa Molina-Vega, Rachel Pferdehirt, Andrew J Vardanian

**Affiliations:** David Geffen School of Medicine at University of California Los Angeles, Los Angeles, CA; Division of Plastic and Reconstructive Surgery, University of California Los Angeles, Los Angeles, CA; Division of Plastic and Reconstructive Surgery, Keck School of Medicine, University of Southern California, Los Angeles, CA

## Abstract

**Introduction:**

Hyperbaric oxygen therapy (HBOT) involves inhaling 100% oxygen at pressures above one atmosphere absolute in a specialized chamber. HBOT has been used across multiple medical fields and proposed as an adjunctive treatment to enhance healing and clinical outcomes in patients with burns. Despite increasing interest and utilization, the efficacy and benefits of HBOT in burn management remain inconsistent, with no clear consensus established in clinical practice.

**Methods:**

A comprehensive literature search was conducted in September 2025 across three electronic databases: PubMed, Cochrane Library, and Embase, with no restrictions on publication date. The search strategy aimed to identify studies evaluating the use HBOT for the treatment of burns. Inclusion criteria were human studies, articles published in English, and availability of full text. The selection process adhered to the Preferred Reporting Items for Systematic Reviews and Meta-Analyses (PRISMA) guidelines. Two independent reviewers screened titles and abstracts for relevance, with discrepancies resolved through a third reviewer. Eligible studies included those that applied HBOT as a treatment modality for burns and reported clinical outcomes.

**Results:**

The systematic review included a total of 13 studies that met the predefined inclusion criteria, comprising 5 randomized controlled trials, 7 cohort studies, and 1 case–control study. Collectively, these studies evaluated HBOT in a total of 566 burn patients. The study populations exhibited considerable variability in burn severity, HBOT treatment protocols, and outcome measures. Several studies reported that HBOT was associated with reduced hospital length of stay and a decreased need for surgical interventions. Additionally, some evidence indicated that HBOT may enhance wound healing rates and lower the risk of infection. However, results regarding mortality benefits were inconsistent across the included studies. Despite these promising findings, heterogeneity in study design, patient characteristics, and HBOT regimens limited synthesis through meta-analysis.

**Conclusions:**

Hyperbaric oxygen therapy shows potential as an adjunctive treatment for burns by improving wound healing and reducing complications such as infection and hospital stay. However, due to variability in study designs and inconsistent findings, definitive conclusions regarding its impact on mortality and long-term outcomes remain uncertain. Well-designed, large-scale randomized controlled trials are needed to establish standardized protocols and clarify the clinical efficacy of HBOT in burn management.

**Applicability of Research to Practice:**

Until definitive trials, burn centers should recognize the potential beneficial impact of hyperbaric oxygen treatment for management of acute burn injuries and threatened skin grafts. Centers with resources may utilize established HBOT protocols to improve health and recovery in burn injury.

**Funding for the study:**

N/A.

